# Griffithsin tandemers: flexible and potent lectin inhibitors of the human immunodeficiency virus

**DOI:** 10.1186/s12977-014-0127-3

**Published:** 2015-01-23

**Authors:** Tinoush Moulaei, Kabamba B Alexandre, Shilpa R Shenoy, Joel R Meyerson, Lauren RH Krumpe, Brian Constantine, Jennifer Wilson, Robert W Buckheit, James B McMahon, Sriram Subramaniam, Alexander Wlodawer, Barry R O’Keefe

**Affiliations:** Molecular Targets Laboratory, Center for Cancer Research, National Cancer Institute at Frederick, Frederick, MD 21702-1201 USA; Protein Structure Section, Macromolecular Crystallography Laboratory, National Cancer Institute at Frederick, Frederick, MD 21702-1201 USA; Department of Chemistry and Biochemistry, University of Maryland, College Park, MD 20740 USA; Basic Science Program, Leidos Biomedical Research, Inc., Frederick National Laboratory, Frederick, MD 21702 USA; Laboratory of Cell Biology, Center for Cancer Research, National Cancer Institute, Bethesda, MD 20892 USA; Imquest BioSciences, Frederick, MD 21704 USA

**Keywords:** Lectins, HIV, Griffithsin

## Abstract

**Background:**

The lectin griffithsin (GRFT) is a potent antiviral agent capable of prevention and treatment of infections caused by a number of enveloped viruses and is currently under development as an anti-HIV microbicide. In addition to its broad antiviral activity, GRFT is stable at high temperature and at a broad pH range, displays little toxicity and immunogenicity, and is amenable to large-scale manufacturing. Native GRFT is a domain-swapped homodimer that binds to viral envelope glycoproteins and has displayed mid-picomolar activity in cell-based anti-HIV assays. Previously, we have engineered and analyzed several monomeric forms of this lectin (mGRFT) with anti-HIV EC_50_ values ranging up to 323 nM. Based on our previous analysis of mGRFT, we hypothesized that the orientation and spacing of the carbohydrate binding domains GRFT were key to its antiviral activity.

**Results:**

Here we present data on engineered tandem repeats of mGRFT (mGRFT tandemers) with antiviral activity at concentrations as low as one picomolar in whole-cell anti-HIV assays. mGRFT tandemers were analyzed thermodynamically, both individually and in complex with HIV-1 gp120. We also demonstrate by dynamic light scattering and cryo-electron microscopy that mGRFT tandemers do not aggregate HIV virions. This establishes that, although the intra-virion crosslinking of HIV envelope glycoproteins is likely integral to their activity, the antiviral activity of these lectins is not due to virus aggregation caused by inter-virion crosslinking.

**Conclusions:**

The engineered tandemer constructs of mGRFT may provide novel and powerful agents for prevention of infection by HIV and other enveloped viruses.

**Electronic supplementary material:**

The online version of this article (doi:10.1186/s12977-014-0127-3) contains supplementary material, which is available to authorized users.

## Background

The surface glycoproteins of enveloped viruses act as anchors for docking and fusion with the target cell’s membrane [1]. These glycoproteins are the most prominent features of the viral surface that can be recognized within the host cellular background and are targeted for antibody neutralization. Consequently, viruses have evolved a number of strategies for shielding the spike structures formed by their surface glycoproteins. These strategies include restriction of access to conserved structural features through conformational occlusion and oligomerization [2,3], sequence hyper-variability, especially within loops that mask conserved epitopes [4], and extensive posttranslational glycosylation [5].

N-linked carbohydrates compose approximately 50% of the molecular weight of HIV gp120 [6]. This glycan armor hides the underlying protein structures on one face of gp120, whereas the epitopes on the other face of gp120 are masked by the quaternary structure of the trimeric spike [7]. The effectiveness of glycans in this defensive mechanism may in part hinge on the generally weak interactions between proteins and carbohydrates [8]. The glycan modifications of HIV Env are also essential for its folding and function [9,10]. Evolution of mutations in the position and frequency of N-linked glycans on the HIV spike is a contributing factor to viral escape during the course of infection and appears to occur within limits affected by immune evasion, efficient processing, and access to receptor binding sites [11]. Logically, mu that would reduce the glycosylation levels of HIV Env could affect viral processing and maturation, leading to attenuated infectivity, as well as further exposing the virus to the immune system. Therefore, targeting this viral glycan shield may be an effective way of combating HIV infection.

**Figure 1:**
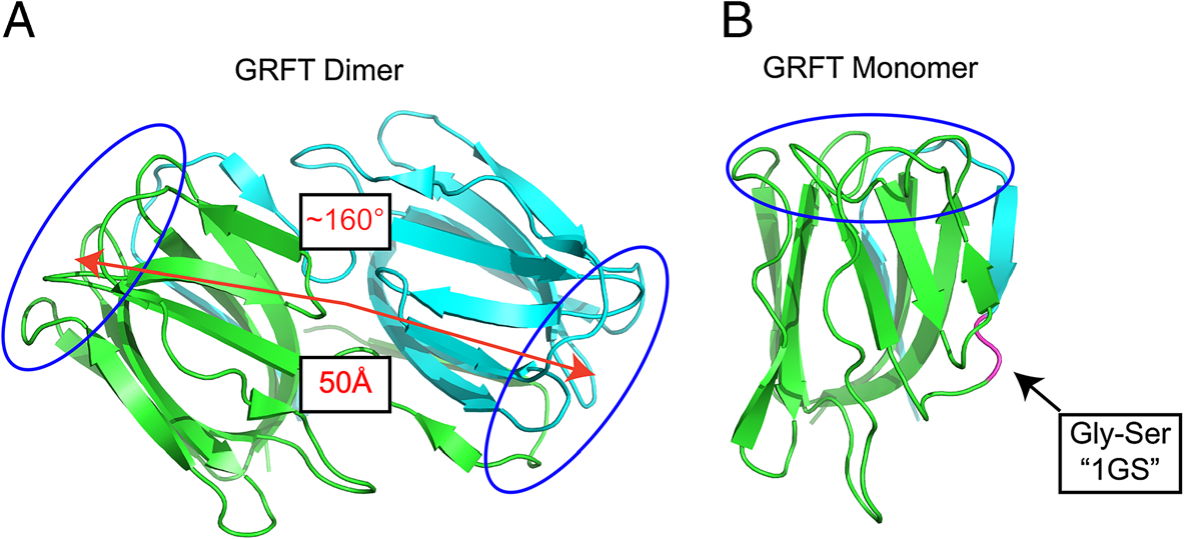
**Structure and binding site orientation of dimeric GRFT and mutations used to generate monomeric GRFT.** Dimeric GRFT **(A)** is a domain-swapped dimer with two identical carbohydrate-binding domains (circled in blue) separated by 50 Å and at a relative angle of ~160° from each other. Obligate monomeric GRFT **(B)** was generated by the addition of Gly-Ser residues in the hinge region of wild-type GRFT. The enhanced flexibility of the hinge region resulted in the collapse of the swapped domain to form an obligate monomer, mGRFT.

Lectins are small proteins that have evolved to bind carbohydrates with high affinity and specificity. A number of lectins have been shown to display potent antiviral activity [12]. Currently, the most potent reported anti-HIV lectin is griffithsin (GRFT), an obligate domain-swapped dimer in which each domain has jacalin-like fold (Figure 1A) [13]. GRFT has anti-HIV EC_50_ of ~50 pM in cell-based assays [14] and exhibits synergistic effects against HIV in combination with other antiviral agents [15-17]. Additionally, GRFT has been reported to exhibit antiviral activity against the coronavirus responsible for SARS [18,19], the hepatitis C virus [20], the Japanese encephalitis virus [21], and the murine herpes simplex virus type 2 [22]. There are a number of characteristics that make GRFT an attractive candidate for development as an antiviral therapeutic. GRFT is thermostable, can survive in a wide range of conditions including those found in the macaque vaginal environment, and exhibits little or no toxicity and immunogenicity [23]. Subcutaneous administration of GRFT in rodent models has also shown that this lectin accumulates to therapeutically effective levels in serum and plasma with minimal toxicity [24]. Finally, low-cost, large-scale production of GRFT in genetically modified tobacco plants has been demonstrated [25].

We have previously determined the structures of unliganded, native GRFT (Figure 1A) and its complexes with a number of mono- and disaccharides [26-28]. In its native form GRFT is a rigid domain-swapped homodimer. We also engineered several monomeric forms of GRFT (mGRFT) (Figure 1B) and determined their structures, including the structure of a complex with nonamannoside, an analogue of Man9 that forms a common glycosylation pattern found on HIV surface glycoproteins [29]. The anti-HIV activity of mGRFT was approximately 1000-fold lower than that of GRFT, although both the monomeric and dimeric forms of this lectin have very similar oligosaccharide-binding affinities. Therefore, we suggested that the presence of two binding domains in native, dimeric GRFT was important for its activity by allowing the cross-linking of gp120-associated oligosaccharides. Such a mechanism could also lead to the agglutination of HIV virions [30]. We hypothesized that providing more flexibility and varying the distance between the two domains might improve antiviral properties of the derivatives of GRFT. Here we describe the design and characterization of four new lectins constructed from two, three, and four tandem repeats of mGRFT (mGRFT tandemers), which allow for variation in the distance between the carbohydrate binding domains and provide significant freedom to their orientation angle. These novel mGRFT tandemers displayed potent anti-HIV activity down to a level of 1.0 pM and were able to do so without engendering viral aggregation.

## Results

### Cloning, expression, and purification of mGRFT and GRFT tandemers

**Figure 2:**
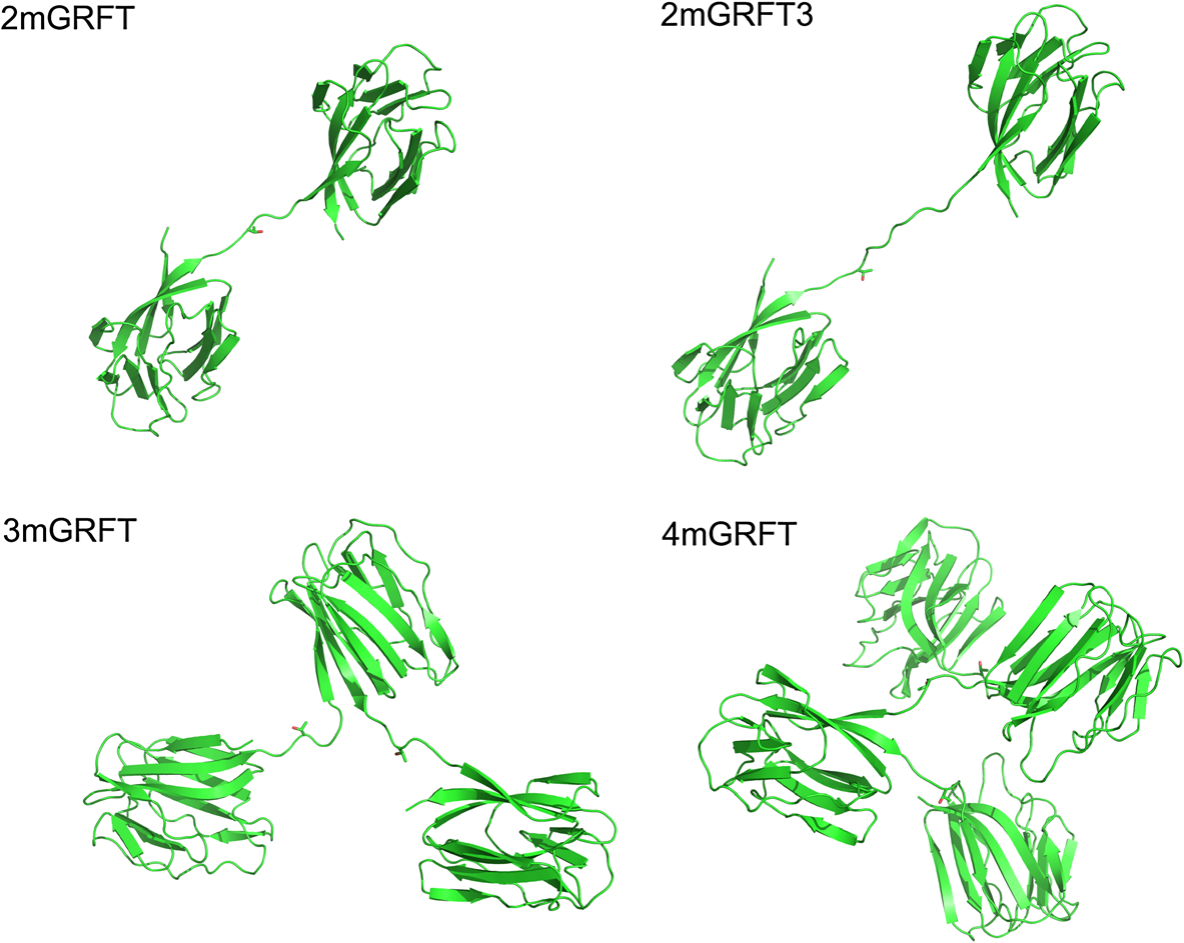
**Theoretical structures of GRFT-tandemers.** Griffithsin tandemers 2mGRFT, 2mGRFT3, 3mGRFT, and 4mGRFT are shown as models of monomeric units attached by flexible linker regions. The N and C termini (red and black, respectively) in a single mGRFT domain are approximately 10 Å apart, causing the individual domains in the tandemers to branch out. Each Gly-Thr-Gly linker (blue) is also approximately 10 Å long in its extended conformation.

The GRFT monomer 1GS-S ([30], PDB ID 3LL2, Figure 1B) was chosen as the repeating unit in the design of mGRFT tandemers, because the L2S mutation at its N terminus rendered this monomer more susceptible to proteolytic cleavage of its N-terminal affinity tag. All further references to mGRFT in this work are to 1GS-S. Schematic models of mGRFT tandemers constructed based on the structure of mGRFT [30] are depicted in Figure 2. The linker between each mGRFT domain was chosen to be flexible and unstructured and consisted of single and triple repeats of Gly-Thr-Gly. However, the flexibility of the linker rendered all attempts at crystallizing the tandemers unsuccessful. Purification of tandemers was performed largely as described previously [30]. The relevant information for GRFT, mGRFT, and the tandemers is listed in Table 1. The relative thermal stability of the mGRFT tandemers was evaluated by differential scanning calorimetry (Additional file 1: Table S1). It was found that the mGRFT tandemers have similar melting temperatures to mGRFT, indicating that the lectin domains were properly folded.

**Table 1:** **Physical characteristics an anti-HIV activity of griffithsin tandemers**

**Name**	**Number of domains**	**Linker**	**Molecular Weight (kDa)**	**Anti-HIV EC**_**50**_^**1**^ **(pM)**	**Terminal binding surface distance (Å)**
GRFT	**2**	**none**	**25.6**	**13.8 ± 0.3**	**50**
mGRFT (1GS-S [30])	**1**	**none**	**12.8**	**119,300 ± 19,300**	**n.a.**
2mGRFT	**2**	**Gly-Thr-Gly**	**25.8**	**2.7 ± 2.7**	**70**
2mGRFT3	**2**	**(Gly-Thr-Gly)**_**3**_	**26.2**	**2.6 ± 5.3**	**90**
3mGRFT	**3**	**Gly-Thr-Gly**	**38.8**	**1.0 ± 2.9**	**90**
4mGRFT	**4**	**Gly-Thr-Gly**	**51.8**	**1.2 ± 0.3**	**100**

^1^Effective concentration at which 50% of CEM-SS cells are protected from the cytopathic effects of HIV-1_RF_ infection.

### Anti-HIV cytopathicity assay

The mGRFT tandemers, as well as mGRFT and GRFT, were tested simultaneously in a whole-cell anti-HIV assay that measures HIV-1_RF_-induced cytopathicity in the T-lymphoblastic cell line CEM-SS. This assay also tests for compound-induced cytotoxicity to uninfected CEM-SS cells. The results (Table 1) showed that, as reported previously, mGRFT was significantly weaker than GRFT, with an EC_50_ value of 119.3 nM. For the mGRFT tandemers, 2mGRFT (EC_50_ = 2.7 pM) and 2mGRFT3 (EC_50_ = 2.6 pM) were 5-fold more active than GRFT (EC_50_ = 13.8 pM). The antiviral activity displayed by the 3mGRFT tandemer was enhanced by another 5 fold, with an EC_50_ = 1.0 pM. However, no further enhancement of antiviral activity was observed for constructs with additional mGRFT domains, since the anti-HIV activity of 4mGRFT (EC_50_ = 1.2 pM) was similar to 3mGRFT. The similar anti-HIV activity of 2mGRFT and 2mGRFT3 indicated that the longer interdomain linker did not affect the potency of the tandemers. None of the mGRFT tandemers displayed any toxicity to the CEM-SS cells at the highest tested doses (see Additional file 2: Figure S1).

### Single cycle pseudo-HIV-1 virus neutralization assay

All mGRFT tandemers as well as mGRFT and native dimeric GRFT were tested for their activity against a spectrum of HIV-1 strains from different subtypes in a Tzm-bl single cycle neutralization assay system. As expected, the activity of all GRFT molecules tested was reduced when compared to the multiple cycle HIV-1 induced cytopathicity assay. The results, as shown in Table 2, indicate that the 3mGRFT and 4mGRFT tandemers were, on average, more potent than native dimeric GRFT, whereas the 2mGRFT and 2mGRFT3 tandemers were not significantly better than native dimeric GRFT. Strikingly, two viral strains, subtype B CAAN5342.2 and subtype C CAP206.8 were shown to be resistant to native dimeric GRFT but retained sensitivity to the 3mGRFT and 4mGRFT tandemers, with the 3mGRFT and 4mGRFT tandemers at least 10-fold more active than native dimeric GRFT. A similar result was also seen with the subtype A virus Q168.a2 which was also ~10 times more sensitive to the 3mGRFT and 4mGRFT tandemers.

**Table 2:** **Anti-HIV-1 activity of mGRFT tandemers, mGRFT and native dimeric GRFT in a single cycle Tzm-bl pseudovirus assay system**

**Envelope**	**IC50 (nM)**				
	**2MG**	**2MG3**	**3MG**	**4MG**	**GRFT**
**Subtype B**					
PVO.4	0.301±0.0818	0.558±0.105	0.270±0.0344	0.185±0.0151	0.0370±0.0173
QH0692.42	0.202±0.0842	0.513±0.0882	0.223±0.0834	0.170±0.0119	0.0473±0.0152
JR-FL	2.09±3.28	5.77±2.16	0.325±0.0702	0.287±0.0476	0.922±0.354
CAAN5342.A2	5.27±3.28	16.9±1.41	0.326±0.0834	0.232±0.0167	7.34±0.927
*Median*	*1.20*	*3.16*	*0.298*	*0.0208*	*0.485*
**Subtype C**					
Du156.12	0.0182±0.00133	0.0616±0.00335	0.0794±0.0252	0.0887±0.0185	0.0324±0.00204
Du179.14	0.158±0.0962	0.144±0.0438	0.124±0.0262	0.0934±0.0291	0.606±0.139
COT6.15	1.46±0.452	2.54±1.01	0.412±0.0588	0.273±0.0396	0.734±0.326
DU151.2	0.694±0.144	1.70±0.0306	0.308±0.0169	0.300±0.0285	1.49±0.225
CAP206.8	0.178±0.0750	0.696±0.0141	0.181±0.0546	0.164±0.0586	2.14±1.26
*Median*	*0.178*	*0.696*	*0.181*	*0.164*	*0.734*
**Subtype A**					
Q23.7	7.26±0.225	7.16±0.512	0.432±0.101	0.276±0.0710	1.19±0.535
Q168.a2	1.55±0.723	5.28±1.48	0.331±0.0398	0.273±0.0167	3.80±1.03
*Median*	*4.41*	*6.22*	*0.396*	*0.274*	*2.5*

**Figure 3:**
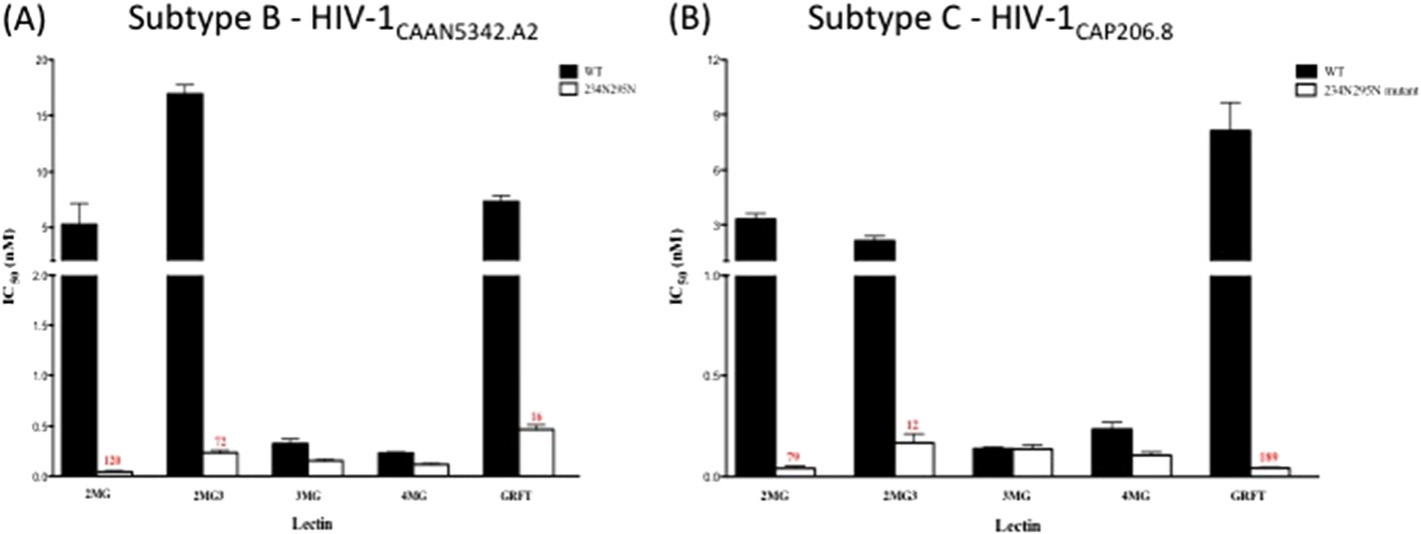
**Effect of the addition of glycans at positions 234 N and 295 N on HIV-1 sensitivity to GRFT and mGRFT-tandemers.** Pseudoviruses from either **(A)** HIV-1 _CAAN5342.A2_ or **(B)** HIV-1 _CAP206.8_ bearing either wild-type gp120 (black bars) or re-engineered mutant gp120 restoring the glycosylation sites at position 234 and 295 (white bars) were tested for their sensitivity to native GRFT or mGRFT tandemers. Anti-HIV activity was visualized using the single-cycle Tzm-bl assay system with activity reported as the effective concentration that inhibited infection by 50% (EC_50_). All experiments were done in triplicate with the average value reported. Numbers in red indicate fold-difference in EC_50_ values between wild-type and mutant gp120 bearing pseudoviruses.

To evaluate the role of specific oligosaccharides in the sensitivity of subtype B CAAN5342.2 and subtype C CAP206.8 to wild type dimeric GRFT compared to the 3mGRFT and 4mGRFT tandemers we undertook to restore two specific oligosaccharide attachment sites on gp120 into these Env constructs by mutating specific residues at positions 234 N and 295 N. As can be seen in Figure 3, insertion of these two oligosaccharide attachment sites restored sensitivity to native dimeric GRFT (as well as the 2mGRFT and 2mGRFT3 tademers) but did not significantly affect the activity of the 3mGRt and 4mGRFT tandemers.

### Isothermal titration calorimetry

Isothermal titration calorimetry was used to characterize the thermodynamics of binding of the mGRFT tandemers to the HIV envelope glycoprotein gp120, as shown previously with GRFT [14]. Since 4mGRFT exhibited no better activity than 3mGRFT in anti-HIV assays, it was not investigated further. The gp120 binding of 2mGRFT, 2mGRFT3, and 3mGRFT was fully characterized and the thermodynamic parameters of the interactions are detailed in Table 3. Binding of gp120 to the mGRFT tandemers was revealed to be exothermic in nature. The exothermic heats of interaction (negative ∆H values; Table 2) were likely due to polar and electrostatic interactions between mGRFT domains and the high-mannose oligosaccharides of gp120. As reported previously for the binding between GRFT and high-mannose oligosacharides [30], an enthalpy-entropy compensation was apparent, indicating that the favorable binding contacts between GRFT and gp120 had overcome the unfavorable conformational penalties of the bound partners and the unfavorable entropy of water (negative T∆S values; Table 3) at the interface and around the bound complex, resulting in energetically-favored binding with gp120 (negative ∆G values; Table 3).

**Table 3:** **Thermodynamic parameters of griffithsin binding to HIV-1 gp120**

	**Affinity (** **μM)**	Δ**H (kcal/mol)**	Δ**G**	**T**Δ**S**	Δ**H/T**Δ**S**
**GRFT**	**0.008 ± 0.004**	**– 30.4 + 0.3**	**– 11.2 ± 0.3**	**– 19.2 ± 0.3**	**1.58**
**mGRFT**	**0.112 ± 0.050**	**– 35.5 + 0.6**	**– 9.5 ± 0.7**	**– 26.0 ± 0.7**	**1.36**
**2mGRFT**	**0.005 ± 0.002**	**– 69.6 + 0.5**	**– 11.6 ± 0.6**	**– 59.9 ± 0.5**	**1.16**
**2mGRFT3**	**0.003 ± 0.001**	**– 47.2 + 0.6**	**– 11.8 ± 0.2**	**– 35.4 ± 0.6**	**1.33**
**3mGRFT**	**0.003 ± 0.001**	**– 74.4 + 0.4**	**– 11.8 ± 0.2**	**– 62.7 ± 0.4**	**1.19**

The observed tight, nanomolar binding affinities were close to the accuracy limits of the calorimetric instrument but well within the experimental error and the calculated K_d_ values (Table 3), showed that the mGRFT tandemers bound gp120 with slightly stronger affinity than GRFT. Particularly in the case of the 2mGRFT3 and 3mGRFT constructs, a 3 nM K_d_ value was calculated for both tandemers. Deconstruction of the gp120 binding affinities into their respective enthalpic and entropic contributions provided further insight into the nature of the binding between the mGRFT tandemers and gp120. From an examination of the enthalpy of binding (ΔH) it was clear that the mGRFT tandemers mediated a higher number of binding contacts with gp120 than did GRFT (Table 3).

The enthalpy of binding was, however, not the sole determinant of an optimal binding interaction. Although 2mGRFT and 3mGRFT binding events were the most exothermic, the 2mGRFT3 construct produced the best overall binding profile among the mGRFT tandemers, showing very little entropic cost for binding (TΔS = − 35 kcal/mol). The enthalpy/entropy compensation (ΔH/ΔS) ratio for 2mGRFT3 was 1.33, compared to 1.16 and 1.19 for 2mGRFT and 3mGRFT, respectively (Table 3). As with any tethered constructs, the crosslinking interactions with gp120 and formation of water filled aggregates would be entropically disfavored, primarily due to the decreased entropy of the “caged” water molecules within the crosslinked complexes [31]. It was clear from the enthalpy-entropy compensation that the mGRFT tandemers either did not form large aggregates with gp120, or if they did, the crosslinked complexes were soluble and flexible enough to prevent any great degree of trapping of the solvent water molecules. The gp120 titrated solutions of the mGRFT tandemers were far less cloudy than the post-titration solution of GRFT, suggesting that little or no crosslinked aggregation had taken place. In particular, solution of the 2mGRFT3 complex with gp120 was essentially clear.

### Dynamic light scattering

**Figure 4:**
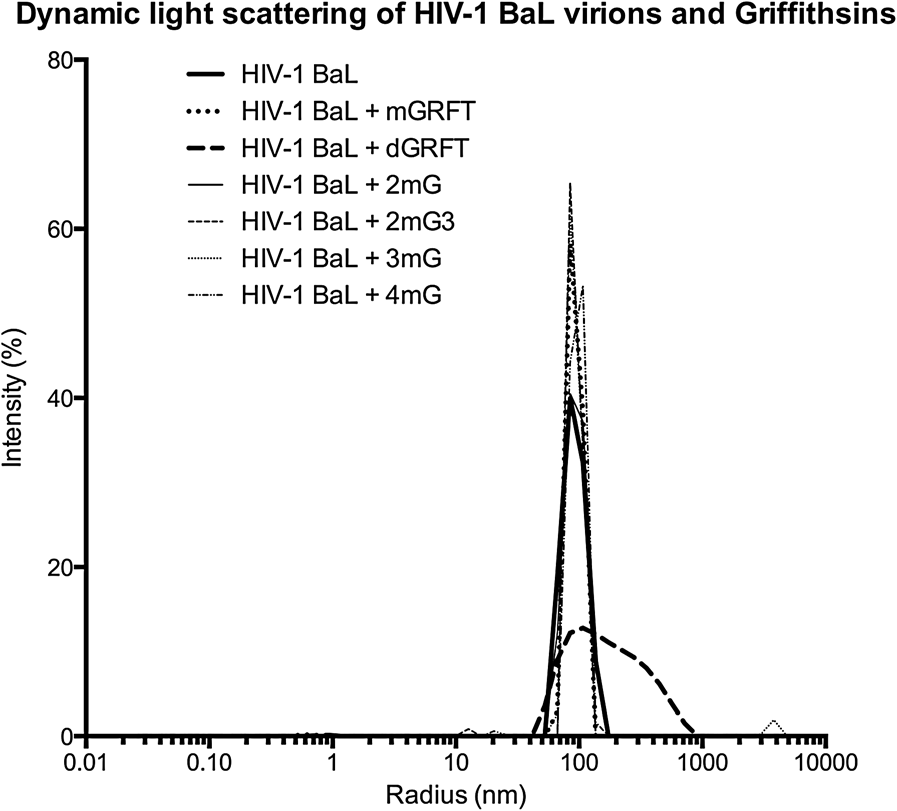
**Results of dynamic light scattering experiments on HIV-1**_**BAL**_ **virions treated with GRFT, mGRFT, or the mGRFT tandemers.** Dynamic light scattering traces are shown for HIV-1_BAL_ viruses without lectin, with mGRFT, with GRFT, or with one of the tandemers. Negative controls with buffers or purified lectin did not measurably scatter light.

One of the common attributes of many lectins is their capacity to agglutinate pathogens [32]. Although antiviral lectins such as GRFT have been shown not to agglutinate human cells [14], it has been suggested that these lectins, which contain multiple binding domains, aggregate viruses [30]. To determine if this was true and to evaluate the role that the enhanced flexibility in binding domains may play in this phenomenon, we used dynamic light scattering to measure the aggregation of HIV-1_BAL_ virions after treatment with mGRFT, GRFT, or one of the four GRFT tandemers. As shown in Figure 4, GRFT did aggregate HIV-1_BAL_ virions to a significant extent when compared to untreated virions. As expected, mGRFT, with only a single binding domain, did not aggregate virions but, interestingly, also none of the GRFT tandemers, including 4mGRFT with its four binding domains, caused virions to aggregate.

### Cryo-electron microscopy

**Figure 5:**
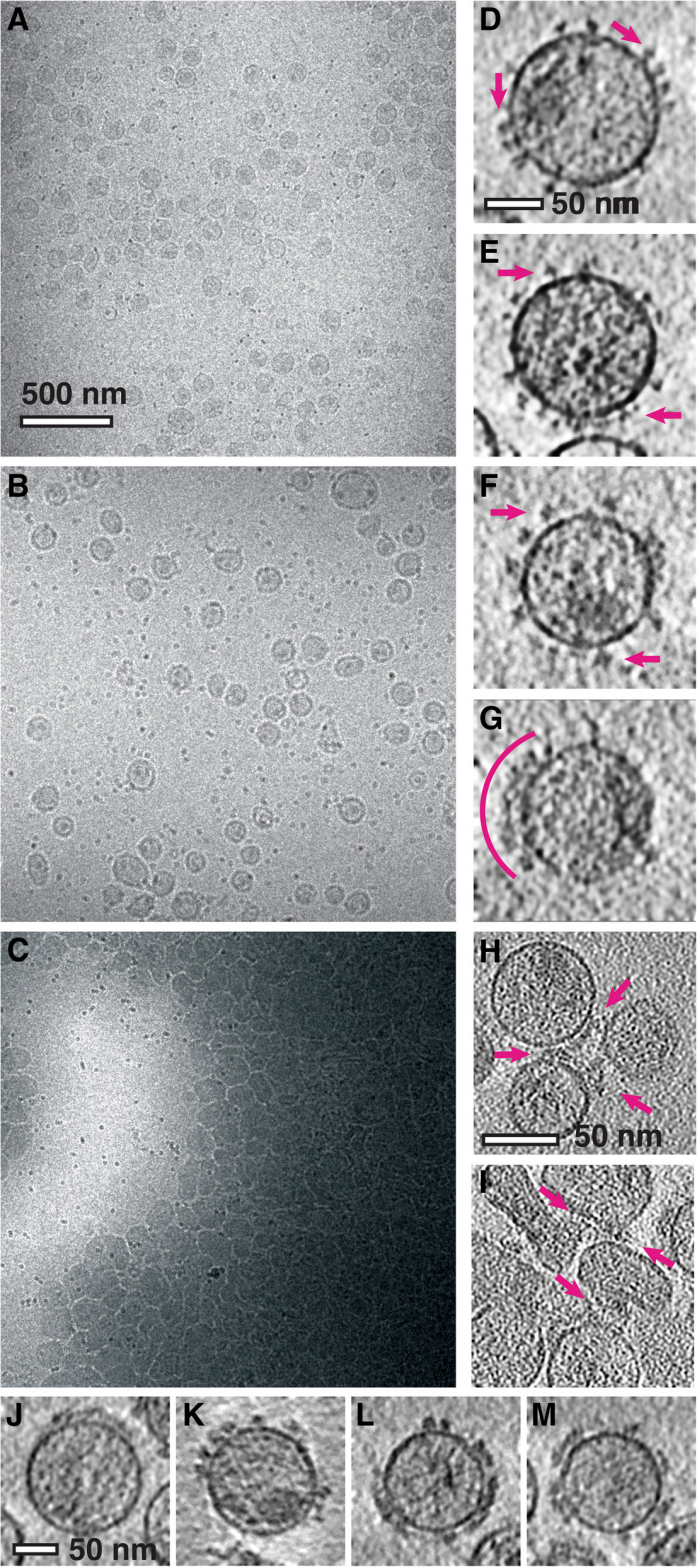
**Electron micrographs of the HIV-1 virions interacting with different constructs of GRFT-based lectins. (A)** Projection of untreated HIV-1 virions. Virions are observed as ~ 100 nm circles in the imaging field which includes 10-nanometer-sized protein-A gold fiducials which appear as dark spots. **(B, C)** Slices through tomograms of untreated HIV-1 virions with red arrows indicating individual Env proteins. **(D)** Projection of HIV-1 virions that were incubated with mGRFT prior to vitrification. **(E, F)** Slices through tomograms of HIV-1 virions treated with mGRFT. Representative Env glycoprotein spikes are indicated by red arrows **(E)**. A patch of glycoprotein spikes is indicated by a red arc **(F)**.** (G)** Projection of HIV-1 virions that were incubated with native GRFT prior to vitrification. **(H, I)** Slices through tomograms of virions at the periphery of the aggregates in virions treated with native GRFT. **(J–M)** Slices through tomograms collected from vitreous preparations of HIV-1 virions with GRFT tandemer constructs, 2mGRFT **(J)**, 2mGRFT3 **(K)**, 3mGRFT **(I)**, and 4mGRFT **(M)**. Scale bars are 500 nm in panels (A, D, and G) and 50 nm in the remaining panels.

The interactions of GRFT, mGRFT, and the tandemers with purified HIV-1_BaL_ virions were evaluated using cryo-electron microscopy. The control experiments with HIV-1_Bal_ in the absence of any lectins showed a uniform distribution of virions in the imaging field, with virions having approximately 100 nm diameter and spherical shape (Figure 5A). Ten-nanometer gold fiducials appear as electron dense spots in the micrographs. When virions were imaged at high magnification with cryo-electron tomography, individual envelope glycoprotein spikes were visible (Figures 5B, C, red arrows). The spikes were of the expected height of ~ 120 Å as measured from the membrane, with a structural profile consistent with previous electron tomographic studies [33-38].

The overall spatial distribution and shape of the virions in the presence of mGRFT appeared similar to that of the negative control (Figure 5D). However, differences in spike size and structural profile were evident (Figure 5E and F, red arrows). Viruses exposed to mGRFT displayed an additional density on the spike that was irregular in both shape and the degree of enlargement. The effect of GRFT on HIV-1 differed dramatically from that of mGRFT. Virions treated with GRFT formed large aggregates having lateral dimensions on the micrometer scale (Figure 5G). These aggregates appear in the micrograph as large electron-dense swathes. Individual virions could be identified at the periphery of the aggregates, and though they seem to maintain their membrane integrity, their shapes were highly distorted. Tomography revealed dense masses of protein at the interfaces between virions (Figures 5H and I), which consistently co-localized to the interface between neighboring virions. The mGRFT tandemers displayed mutually similar effects on HIV-1 virions and envelope glycoproteins (Figures 5J, K, I, and M, respectively), but differed significantly from virions treated with GRFT. Tomographic imaging showed that virions treated with the mGRFT tandemers remained monodisperse, similar to the results with mGRFT and dissimilar from GRFT.

## Discussion

The functional unit of native GRFT is an obligate domain-swapped homodimer [39], with two carbohydrate-binding surfaces located at the opposite ends of the dimeric molecule. The first 15 residues of each monomer form a strand-loop-strand structure that completes the β-prism-II fold of the opposite monomer. Each β-prism harbors three carbohydrate-binding sites on one of the terminal facets of the prism. The centers of the carbohydrate-binding sites form an equilateral triangle with 15 Å sides. The domains are oriented at a ~160° angle from each other, with the centers of the triangular carbohydrate-binding surfaces positioned 50 Å apart (Figure 1A). The orientation of the domains is fixed, since the dimer is formed by domain swapping involving two β strands, resulting in a rigid molecule [26-28] The three carbohydrate-binding sites on the surface of each domain act as a selective mechanism for specifically targeting high-mannose, branched carbohydrate environments typically found on viral envelope glycoproteins [40]. Viral glycoproteins present a preponderance of high mannose oligosaccharides when compared to human cellular glycoproteins, which have a larger proportion of sialylated oligosaccharides. Alignments of glycans identified on gp120 *in vitro* [6,41] with N-linked sequon variations of gp120 identified *in silico* suggests that high-mannose modifications dominate the conserved regions of gp120 [11].

The mGRFT tandemers described here were designed to evaluate the role of the fixed spatial orientation of GRFT on its anti-HIV activity. By allowing for more flexibility in the linker region of the mGRFT tandemer structures we enhanced the ability of the second mGRFT bining site to sample the local oligosaccharide environment on individual virions. The larger mGRFT tandemers (2mGRFT3, 3mGRFT and 4mGRFT) also increased the distance between the individual mGRFT binding domains, thus increasing the potential scope of GRFT-oligosaccharide interactions to distances greater than 50 Å.

All of the designed mGRFT tandemers were readily expressed in *E. coli* and purified to homogeneity. The mGRFT tandemers are distinct from other recently published GRFT tandemer constructs [42] in that they were created using obligate monomeric forms of GRFT [30], thereby preventing any potential for domain swapping by the expressed mGRFT tandemers. The maintenance of the mGRFT form of individual lectin domains in the mGRFT tandemers was confirmed by differential scanning calorimetry which showed that mGRFT tandemers melted at temperatures corresponding to mGRFT and not native, dimeric GRFT (Additional file 1: Table S1).

Previous calorimetric evaluations of the binding of high-mannose oligosaccharides by GRFT [14,18,30] and our present data on the binding of the mGRFT tandemers to gp120 showed an overall similar mode of interaction, primarily involving favorable polar-electrostatic contacts. Clear differences among the antiviral proteins were evident, however, when the bindings were parsed into individual thermodynamic contributions (Table 3). The data suggested that steric hindrance and/or lack of flexibility in GRFT appeared to hamper its domains from making full contact with gp120, whereas the flexible linkers in the GRFT tandemer structures allowed their mGRFT domains to sample more extensively the gp120 surface glycans (comparing ΔH values in Table 3).

The mGRFT constructed in our previous work displayed a large reduction in its anti-HIV activity in the CEM-SS HIV-1-induced cytotoxicity assay system. It is important to note that, whereas mGRFT was far less active than GRFT, its anti-HIV activity was still in the nanomolar range [30]. The mGRFT tandemers created for this study all showed anti-HIV activity significantly better than mGRFT and five- to ten-fold better than native, dimeric GRFT in the same assay system (Table 1). We also tested the mGRFT tandemers in a TzmBl single cycle pseudovirus assay system against several different strains of HIV-1 to assess their broader activity compared to both mGRFT and native GRFT. The results showed that the 3mGRFT and 4mGRFT tandemers had better overall anti-HIV activity than wild-type GRFT with significant strain variance in EC_50_ values (Table 2). The data also showed that the 3mGRFT and 4mGRFT tandemers retained activity against strains of virus which displayed a level of resistance to native dimeric GRFT.

We evaluated the data to determine whether or not increasing the length of inter-domain linkers would affect anti-HIV activity. The maximum theoretical distance between the terminal carbohydrate-binding surfaces of each mGRFT tandemer was calculated based on basic models derived from the X-ray crystal structure of mGRFT (Table 1, Figure 2). In native GRFT the distance between the centers of the carbohydrate-binding regions on each domain was ~50 Å (Figure 1A). Increasing the linear length of the mGRFT tandemers from ~70 Å (2mGRFT) to ~90 Å (2mGRFT3) resulted in a nearly identical or slightly worse anti-HIV activity overall, suggesting that this increase in potential distance between mGRFT domains was not a determining factor in anti-HIV potency. Increasing the number of mGRFT domains did enhance antiviral activity. However, this effect was limited to three mGRFT domains as evidenced from the nearly identical anti-HIV activities of 3mGRFT and 4mGRFT (Tables 1 and 2). The limit on the number of mGRFT domains was possibly due to geometric constraints that could limit access to glycan ligands by the fourth mGRFT domain of 4mGRFT and/or saturation of local viral oligosaccharides with mGRFT domains.

The anti-HIV activity of mGRFT tandemers does not stem from aggregation of virions, as evidenced by the absence of virion agglutination in dynamic light scattering experiments (Figure 4). In contrast, native GRFT binding to HIV-1 resulted in extensive aggregation of virions (Figure 4). The dimeric structure of native GRFT enhanced its anti-HIV activity by orders of magnitude but also led to viral aggregation. While this aggregation is a possible contributing factor to the antiviral activity of native GRFT, the ability to cause aggregation is clearly not the determining factor for the anti-HIV activity of the mGRFT tandemers. This was evident from both the electron microscopy and light scattering results for HIV treated with the four tandemers. All tandemers were equally or more active than GRFT but none of the mGRFT tandemers caused aggregation of HIV virions.

The inability of the mGRFT tandemers to cause viral aggregation likely stems from the conformational flexibility of the linkers between the mGRFT domains. In native GRFT the two domains are rigidly held across from each other. When one domain of GRFT binds to an Env spike, the second domain would be oriented away at a ~160° angle from that spike and is likely more available for binding the glycans on other Env spikes as well as the glycans on the same spike. In the case of the mGRFT tandemers, after binding of the first mGRFT domain to an Env spike, the flexible linkers would allow the remaining mGRFT domains to more readily sample the local environment and bind to the nearest available carbohydrate ligand. The local concentration of ligands available for subsequent mGRFT domains is potentially higher on the same spike than in the overall solution. Therefore, the mGRFT tandemers, owing to the flexibility of their linkers, are more likely to bind glycans on the same virion, whereas native GRFT, due to its conformational rigidity, would more favor inter-virion cross-linking.

**Figure 6:**
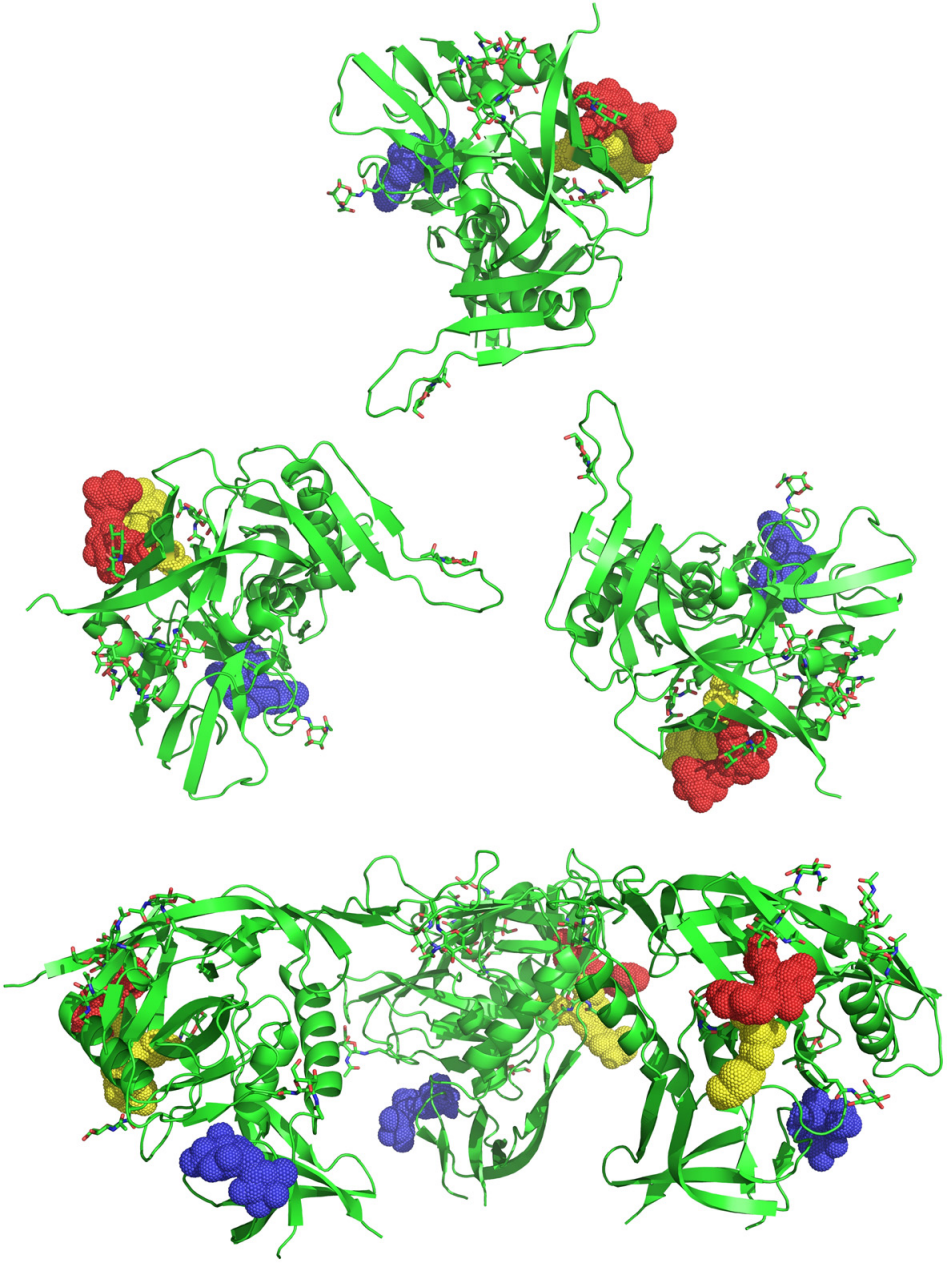
**Location of GRFT binding-associated oligosaccharides (coupled to Asn234, Asn295, and Asn448) in the context of HIV trimeric spike.** Disaccharide modification of Asn234 (blue), Asn295 (red) and Asn448 (yellow) are displayed on a gp120 trimer. X-ray crystal structure of the partially glycosylated HIV-1_HXBc2_ gp120 (PDB 1GC1 [45]) was superimposed on unliganded gp120 electron tomography structure (PDB 3DNN [34]).

These observations have implications for understanding the mechanism whereby the mGRFT tandemers neutralize HIV. The antiviral activity of GRFT, mGRFT, and the mGRFT tandemers stems from their selective binding to high mannose oligosaccharides on Env spikes. Natural mutations that removed glycosylation at positions Asn234 and Asn295 have been reported to impart resistance to GRFT [43]. In addition, Huang *et al.* showed that deglycosylation of gp120 at Asn295 or Asn448 also resulted in resistance to GRFT [44]. The location of these oligosaccharide attachment sites on gp120 are shown in Figure 6. To investigate the role of specific oligosaccharides in the sensitivity to GRFT tandemers, we utilized genetically-engineered pseudotype viruses including either HIV-1 clade B CAAN5342 gp120 (Figure 3A) or clade C CAP206.8 gp120 (Figure 3B) sequences. We tested both strains in either the presence or absence of oligosaccharide attachment sites at positions 234 N and 295 N of the gp120 protein. Figure 3 displays the results of our assessment of the activity of either wild-type GRFT, mGRFT, or mGRFT tandemers against these viruses. The 3mGRFT and 4mGRFT tandemers retain significantly more activity against wild-type GRFT-resistant viruses than the 2mGRFT, 2mGRFT3, or wild-type GRFT. We speculate that this increased activity against resistant viruses is due to the ability of these larger mGRFT tandemers to sample a broader range of oligosaccharides on gp120 than the smaller mGRFT tandemers or the conformationally-restricted wild-type GRFT.

Mapping the location of the Asn234, Asn295, and Asn448 glycans on trimeric spike structures (Figure 6) suggests that glycans decorating these asparagines are located on the lateral edges of HIV spikes. The arrangement of the griffithsin monomers in the mGRFT tandemers is such that they are able to bind carbohydrate moieties on the same virion, but not in a way that bridges separate virions, as demonstrated by the tomographic and light scattering experiments. Given the molecular dimensions of the mGRFT tandemers (<100 Å across), and the fact that neighboring spikes on the virus are spaced apart by significantly greater and variable distances (each trimeric Env itself is ~ 150 Å across), it follows that the multiple sites on the mGRFT tandemers are occupied by carbohydrates that are displayed within the same Env trimer, with a high likelihood of crosslinking across protomers within the trimer. Cryo-electron microscopic studies have shown that CD4-induced opening of the trimeric Env spike is necessary for exposure of gp41 that initiates the first step in the fusion of viral and target cell membranes [34,36,46]. The crosslinking of gp120 protomers may essentially block the opening of the trimeric spike and prevent exposure of gp41 and its fusogenic components required for viral entry, thus providing a likely mechanism for the potent function of GRFT and mGRFT tandemers as antiviral agents.

## Conclusion

The mGRFT tandemers display picomolar anti-HIV activity and, unlike native GRFT, do not aggregate viral particles. mGRFT tandemers also retain potency against viruses resistant to native GRFT. The data presented here demonstrate the potential utility of mGRFT tandemers for the microbicide development, as well as highlight the complex spatial and orientation requirements for viral neutralization by carbohydrate-binding agents such as griffithsin. The 3mGRFTand 4mGRFT tandemers display some advantages over native GRFT (*e.g.* activity against GRFT-resistant viruses) but the addition of flexible linkers makes these constructs more susceptible to proteolytic degradation and less thermally stable. The advantages or disadvantages of the absence of viral aggregation by mGRFT tandemers, though perhaps responsible for their moderately improved *in vitro* activity, is of undetermined value in an *in vivo* setting. Further studies on the full physiological and immunological consequences of mGRFT tandemer administration will be necessary to fully evaluate the potential of these engineered lectins for microbicide development.

## Methods

### Cloning, expression and purification of GRFT, mGRFT and mGRFT tandemers

All restriction enzymes were purchased from New England Biolabs. All chemicals were obtained from American Bioanalytical, unless otherwise stated. All primers were purchased from Integrated DNA Technologies. Protein purification was performed as described previously for the monomeric forms of GRFT [32], except that buffer A contained 50 mM maltose.

### Construction of plasmid p420TG and p2mGRFT

For the construction of the mGRFT tandemers we chose a construct (1GS-S) that, as reported previously [30], produced obligate monomeric GRFT. An expression vector containing the gene for 1GS-S (see Ref. [30] for nomenclature of the different constructs of mGRFT) downstream of a TEV protease cleavage site [30] was used as a template to amplify a second 1GS-S cassette using standard PCR protocols and PfuUltra® Hotstart PCR Master Mix (Stratagene). The amplified cassette contained only the 1GS-S gene terminated with a stop codon and flanked by two XhoI restriction endonuclease sites. The amplified 1GS-S cassette was inserted at the XhoI site downstream of the first 1GS-S cassette in the original expression vector used as a template. The correct orientation of the second cassette was verified by sequencing. Site-directed mutagenesis was used to mutate the DNA sequence between the two 1GS-S cassettes to 5’-ggtaccgcgggctagcatatgtcgaccggt-3’, removing the stop codon at the 3’-terminus of the first 1GS-S cassette and introducing a new multiple-cloning site (MCS) with flanking KpnI and AgeI sites (p420TG). In order to create an expression plasmid for 2mGRFT (p2mGRFT), site-directed mutagenesis was used to alter the MCS in p420TG to 5’-ggtacaggt-3’. The resulting vector contained a single ORF expressing two 1GS-S domains preceded by a TEV protease cleavage site and linked by a GlyThrGly linker.

### Construction of plasmids p3mGRFT, p4mGRFT, and p2mGRFT^long^

Primers 5’- ggaccggtgccgtactgttcatagtagatgtccaggctatc-3’ and 5’-ggggtaccggcagctcgacccatcgcaag-3’ were used with p2mGRFT as the template in a standard PCR reaction. Two amplicons corresponding to single and double 1GS-S cassettes were separated and purified from an agarose gel. Each amplicon contained a KpnI site followed by a glycine codon (ggc) at the 5’ terminus and the same glycine codon followed by a AgeI site at the 3’ terminus.

The single and double 1GS-S cassettes were ligated in between the KpnI and AgeI sites in p420TG to yield p3mGRFT and p4mGRFT, respectively. Site-directed mutagenesis was used to alter the GlyThrGly linker in p2mGRFT to a GlyThrGlyGlyThrGlyGlyThrGly linker, yielding p2mGRFT3 for expression of 2mGRFT3. The complete DNA and amino acid sequences of the GRFT tandemers are included in Additional file 3.

### Purification protocol for tandemers

All tandemers used in the experiments outlined in this paper were purified generally as described previously for the monomeric forms of GRFT [30], except that the cell lysis buffer contained 50 mM maltose to improve recovery. Briefly, cells were lysed in 50 mM Tris (pH 8.0), 200 mM NaCl, 50 mM maltose and 5% v/v BugBuster 10x Protein Extraction Reagent (Merck), with continuous stirring at room temperature overnight. The lysate was then purified by either (i) heating at 50°C for 10 minutes, (ii) adjusting the buffer to 15% v/v ethanol, or (iii) adjusting the buffer to 10% v/v isopropanol. The lysate was centrifuged at 17,000 rpm in a SS-34 rotor for 30 min. The supernatant was loaded onto a Ni-NTA Superflow column (QIAGEN), equilibrated with five column volumes of buffer A (20 mM Tris [pH 8.0], 200 mM NaCl). The column was washed with buffer A and eluted with buffer A containing 250 mM imidazole. TEV protease was added in 1:100 molar ratio of protease to eluted protein and the sample was incubated at room temperature for 1 hr. The TEV-digested sample was then passed over the same Ni-NTA Superflow column equilibrated with five column volumes of buffer A. The flow-through was collected. For further purification the pooled fractions were loaded onto an amylose column (New England Biolabs) equilibrated with five column volumes of buffer A. The column was washed with buffer A and eluted with buffer A additionally containing 50 mM maltose. The pooled fractions containing tandemers were then dialyzed against pure deionized water, or against 20 mM Tris (pH 8.0), 100 mM NaCl. All purification procedures were performed at room temperature. As with the native GRFT, the GRFT tandemers were all stable to pH ranges from 1–8 and to both heat (up to 50°C) and organic solvents (*i.e.* alcohols, acetonitrile).

### Differential scanning calorimetry

Differential scanning calorimetry (DSC) experiments were carried out with a Microcal VP-DSC microcalorimeter (Microcal, Northampton, MA). The concentrations of all tandemer proteins were determined by amino acid analysis, and a 60 μM concentrated sample of a tandemer protein was evaluated per experiment. By routine protocol, buffer (50 mM Tris, 60 mM NaCl) was introduced to both the reference and sample cells and the calorimeter was allowed to ramp through one cycle of a heat-cool cycle (10°C to 90°C) at a heating/cooling rate of 60°C/hr. During the down scan at 25°C, the buffer solution from the sample cell was quickly and efficiently replaced with a degassed tandemer protein sample. The entire system was re-pressurized to approximately 30 psi of positive pressure to prevent evaporation at higher temperatures, and the experiment was allowed to continue. A total of 6 alternating up-down scans (10°C to 90°C) were performed to measure possible reversibility of folding/unfolding of the tandemers. According to manufacturing protocol, Origin DSC Analysis software was used to correct for buffer effects and to carry out the integration of the unfolding transitions of the tandemers. The baseline corrected thermograms were fitted to a two-state melting model and the calorimetric transition enthalpy (ΔH_unf_) was obtained from the area under the excess heat capacity peak, the midpoint of the transition calculated as the melting temperature (Tm).

### HIV-induced cytotoxicity bioassay

A 2,3-bis-[2-methoxy-4-nitro-5-sulfophenyl]-2H-tetrazolium-5-carboxanilide inner salt (XTT)-tetrazolium-based assay was used to determine the anti-HIV activity of mGRFT, GRFT, and mGRFT tandemers against HIV-1_RF_ challenged T-lymphoblastic CEM-SS cells, as described previously [47]. XTT was graciously supplied by the Drug Synthesis and Chemistry Branch, Developmental Therapeutics Program, Division of Cancer Treatment and Diagnosis, National Cancer Institute. CEM-SS cells were maintained in RPMI 1640 media without phenol red and supplemented with 5% fetal bovine serum (BioWhittaker), 2 mM L-glutamine (BioWhittaker), and 50 μg/ml gentamicin (BioWhittaker) (complete medium). Exponentially growing cells were washed and resuspended in complete medium, and a 50 μl aliquot containing 5 × 10^3^ cells was added to individual wells of a 96-well round-bottom microtiter plate containing serial dilutions of GRFT, mGRFT or the mGRFT tandemers (2mGRFT, 2mGRFT3, 3mGRFT, 4mGRFT) in a volume of 100 μl of medium. Stock supernatants of HIV-1_RF_ were diluted in complete medium to yield sufficient cytopathicity (80–90% cell kill in 6 days), and a 50 μl aliquot was added to appropriate wells. Plates were incubated for 6 days at 37°C and then stained for cellular viability using XTT. All experiments were performed in triplicate. To assess activity EC_50_ values were determined by calculating the point at which the dose–response sigmoid curve of increasing cell-survival crossed the point at which 50% of the cells survived. Compound-induced direct cytotoxicity to CEM-SS cells was tested simultaneously by adding identical concentrations of test compounds to wells containing only CEM-SS cells and no virus. Cell viability in these wells was measured at the same time point and by the same methodology as employed for wells used to evaluate the protection against-HIV-1-induced cytopathicity.

### Single-cycle Tzm-bl HIV-1 pseudovirus assay

The single-cycle pseudovirus assay was performed as previously reported using virus strains as described in Alexandre *et al.* [43]. Briefly, HIV-1 psudoviruses were generated using pSG3ΔEnv plasmids, which were co-transfected with various Env from different virus strains into 293 T cells using the Fugene transfection reagent (Roche Applied Sciences, Indianapolis, IN).

The single cycle pseudovirus neutralization assay was performed in a manner similar to that previously reported by Montefiori [48]. Briefly, various dilutions of each compound (GRFT, mGRFT, or mGRFT tandemers) were added to 100 μL of DMEM media augmented with 10% FBS in individual wells of a 96-well plate. To each test well 200 TCID_50_ of pseudovirus in 50 μL of media was added an incubated for 1 hr at 37°C. Then, 100 μL of TZM-bl cells at a concentration of 1×10^5^ cells/mL was added and cultured at 37°C for an additional 48 hrs. Viral infections were visualized by measuring the activity of firefly luciferase. Inhibitory concentrations (IC_50_) were calculated as the concentration resulting in a 50% reduction of relative light units (RLU) compared to virus controls. All measurements were performed in triplicate.

### Isothermal titration calorimetry

Isothermal titration calorimetry (ITC) experiments were performed on a Microcal VP-ITC microcalorimeter (MicroCal, Northampton, MA). In a typical experiment with the mGRFT tandemers and monomeric, glycosylated, bacculovirus-produced HIV-1_IIIB_ gp120 (Immunodiagnostics, Inc., Woburn, MA), the mGRFT tandemer protein (180 μM) was placed in the syringe injector and the gp120 was placed in the calorimeter cell (2.5 μM). In all experiments, a total of 55 injections of tandemer (5 μl/injection) were made, with 600 s spacing between injections. The titrations were all done in a rapidly stirring solution (300 rpm) held at a constant temperature of 30°C. The heats of binding were recorded as the excess power compensation required for maintaining the same temperature during the course of the titration. Baseline experiments of tandemer titration into buffer were done to calculate heats of dilution and this value was subtracted from the experimental heats of binding. The resulting isotherms were fitted using Origin 5.0 nonlinear least-squares program according to manufacturer’s protocol, and the values for the enthalpy of binding (ΔH) and the dissociation constant were obtained. From the dissociation constant, a value for the free energy of binding (ΔG) was extrapolated (ΔG = −RTlnKa), and from this value, the entropy of binding (ΔS) was lastly calculated (ΔG = ΔH –TΔS).

### Dynamic light scattering

Whole HIV-1 viruses (AIDS and Cancer Virus Program, Leidos Inc., Frederick National Laboratory for Cancer Research, Frederick, MD 21702, USA) at stock concentration (~ 10^11^ virions/mL) were diluted 1,000-fold in dilution buffer (10 mM Tris, 150 mM NaCl, 1 mM EDTA, pH 7.4), and 1 mL of this diluted virus volume was transferred to a plastic cuvette. Autocorrelation measurements were carried out at 25°C using a DynaPro NanoStar instrument (Wyatt Technology, Santa Barbara, CA 93117, USA) that provided particle size distributions, the peak of which was taken to be the mean particle size. For dynamic light scattering (DLS) experiments, the viruses were pre-mixed with lectin at the same concentrations used in cryo-electron microscopy experiments. This ensured a valid comparison between dynamic light scattering experiments, and the imaging experiments in which there was no sample dilution before mixing. Dynamic light scattering measurements were carried out with the assistance from Dr. Grzegorz Piszczek (National Institutes of Health, National Heart, Lung and Blood Institute, Biophysics Facility, Bethesda, MD 20814, USA).

### Cryo-electron microscopy

Imaging experiments used purified suspensions of HIV-1_BaL_ virions with estimated concentration of ~ 10^11^ virions/mL (AIDS and Cancer Virus Program, Leidos Inc., Frederick National Laboratory for Cancer Research, Frederick, MD 21702, USA). Prior to use, viruses were inactivated with Aldrithiol-2 which preserves viral entry capacity and antigenic integrity at levels similar to those of untreated virus. Sample mixtures were prepared by adding 10 nm protein-A gold colloid (Cell Microscopy Center, Utrecht University, 3584 CH Utrecht, The Netherlands) to virus suspension, followed by addition of one of six griffithsin constructs and incubation at 4°C for 30 min. All GRFT constructs were added to the virion suspension at equimolar concentrations with respect to the griffithsin monomer. Two microliters of sample mixture were applied to plasma cleaned carbon-coated 200-mesh grids (Quantifoil Micro Tools, 07745 Jena, Germany) and immediately blotted and plunge frozen using a Mark III Vitrobot (FEI Company, Hillsboro, Oregon 97124, USA) maintained at 25°C and 100% humidity. Data was collected on samples maintained at −193°C using a Tecnai G2 Polara transmission electron microscopy (FEI Company, Hillsboro, Oregon 97124, USA) operated at 200 kV and equipped with an energy-filter and 2 K x 2 K post-energy filter CCD camera (Gatan Incorporated, Pleasanton, CA 94588, USA). Projections were acquired with a 10–20 e^−^/Å^2^ dose at 4.5 kX magnification with −70 μm underfocus. Tilt series spanned an angular range of +/− 65° with 2° tilt increments and were acquired at −2.5 μm underfocus with a per-tilt dose of 1 – 2 e^−^/Å^2^. Tilt series were aligned using RAPTOR as implemented in IMOD [49,50], and tomograms were reconstructed using R-weighted back projection as implemented in IMOD.

## Additional files

Additional file 1: Table S1. Differential scanning calorimetry – determination of the melting temperatures (Tm) of griffithsin tandemers. Additional file 2: Figure S1. Dose-response curves for mGRFT tandemers anti-HIV activity. Additional file 3:
**Supplemental Data: Moulaei et al.**
