# Supplementary material for: Griffithsin tandemers: flexible and potent lectin inhibitors of the human immunodeficiency virus
Source: Retrovirology. 2015 Jan 23;12:6. doi: 10.1186/s12977-014-0127-3 (PMC4419512; doi:10.1186/s12977-014-0127-3)
Supplement: Additional file 1: Table S1. — Differential scanning calorimetry – determination of the melting temperatures (Tm) of griffithsin tandemers. [file 12977_2014_127_MOESM1_ESM.docx]

**Supplemental Table 1: Differential scanning calorimetry – determination of the melting temperatures (T_m_) of griffithsin tandemers.**

|  | **T_m_**  **(˚C)** |
| --- | --- |
| GRFT^(a)^ | 78.8 + 0.0 |
| **mGRFT (1GS-S)^(a)^** | 63.5 + 0.1 |
| **2mGRFT** | 61.7 + 0.1 |
|  |  |
| **2mGRFT3** | 61.3 + 0.1 |
|  |  |
| **3mGRFT** | 58.5 + 0.9 |
|  |  |
|  |  |
| **4mGRFT** | 59.1 + 1.1 |
|  |  |
|  |  |
|  |  |

^a^From previously published data: Moulaei T, Shenoy SR, Giomarelli B, Thomas C, McMahon JB, Dauter Z, et al. Monomerization of viral entry inhibitor griffithsin elucidates the relationship between multivalent binding to carbohydrates and anti-HIV activity. Structure. 2010;18:1104-15.
