# Supplementary figures and images for: Griffithsin tandemers: flexible and potent lectin inhibitors of the human immunodeficiency virus
Source: Retrovirology. 2015 Jan 23;12:6. doi: 10.1186/s12977-014-0127-3 (PMC4419512; doi:10.1186/s12977-014-0127-3)

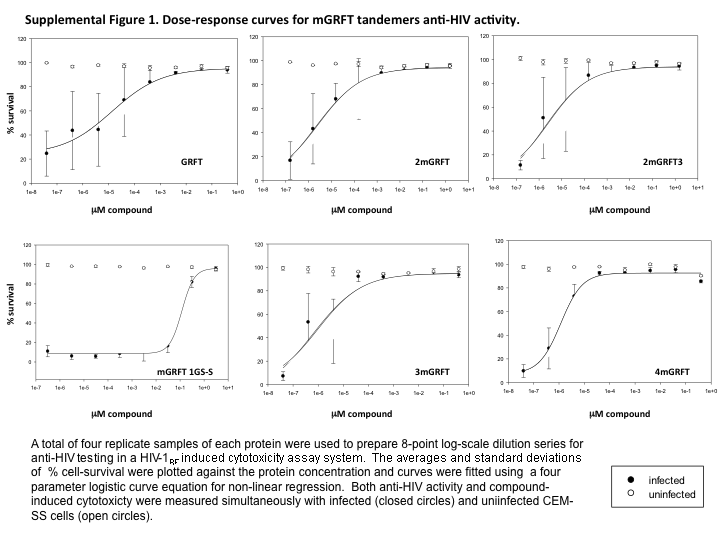

Supplement: Additional file 2: Figure S1. — Dose-response curves for mGRFT tandemers anti-HIV activity. [file 12977_2014_127_MOESM2_ESM.tiff]
