# Supplementary material for: Griffithsin tandemers: flexible and potent lectin inhibitors of the human immunodeficiency virus
Source: Retrovirology. 2015 Jan 23;12:6. doi: 10.1186/s12977-014-0127-3 (PMC4419512; doi:10.1186/s12977-014-0127-3)
Supplement: Additional file 3: — Supplemental Data: Moulaei et al. [file 12977_2014_127_MOESM3_ESM.docx]

**Supplemental Data: Moulaei et al.**

**GRFT tandemer sequences**

2mGRFT

mGRFT-GlyThrGly-mGRFT

DNA sequence:

agctcgacccatcgcaagttcggtggtagtggtggaagtccgttctctggtagcggtctgagcagcattgcagttcgtagtggcagctatctggatgcgatcatcattgatggtgtacatcacggtggctctggtggtaacctgagtccgaccttcacctttggatccggtgagtacatcagcaacatgaccattcgtagtggagactacattgacaacatcagctttgaaaccaacatgggtcgtcgctttggtccgtatggtggatctggtggcagtgcaaacaccctgagcaacgtgaaagtcatccagatcaacggtagtgcaggtgactatctggatagcctggacatctactatgaacagtacggtacaggttcttcgacccatcgcaagttcggtggtagtggtggaagtccgttctctggtagcggtctgagcagcattgcagttcgtagtggcagctatctggatgcgatcatcattgatggtgtacatcacggtggctctggtggtaacctgagtccgaccttcacctttggatccggtgagtacatcagcaacatgaccattcgtagtggagactacattgacaacatcagctttgaaaccaacatgggtcgtcgctttggtccgtatggtggatctggtggcagtgcaaacaccctgagcaacgtgaaagtcatccagatcaacggtagtgcaggtgactatctggatagcctggacatctactatgaacagtactaa

Primary structure:

SSTHRKFGGSGGSPFSGSGLSSIAVRSGSYLDAIIIDGVHHGGSGGNLSPTFTFGSGEYISNMTIRSGDYIDNISFETNMGRRFGPYGGSGGSANTLSNVKVIQINGSAGDYLDSLDIYYEQYGTGSSTHRKFGGSGGSPFSGSGLSSIAVRSGSYLDAIIIDGVHHGGSGGNLSPTFTFGSGEYISNMTIRSGDYIDNISFETNMGRRFGPYGGSGGSANTLSNVKVIQINGSAGDYLDSLDIYYEQY*

3mGRFT

mGRFT-GlyThrGly-mGRFT-GlyThrGly-mGRFT

DNA sequence:

agctcgacccatcgcaagttcggtggtagtggtggaagtccgttctctggtagcggtctgagcagcattgcagttcgtagtggcagctatctggatgcgatcatcattgatggtgtacatcacggtggctctggtggtaacctgagtccgaccttcacctttggatccggtgagtacatcagcaacatgaccattcgtagtggagactacattgacaacatcagctttgaaaccaacatgggtcgtcgctttggtccgtatggtggatctggtggcagtgcaaacaccctgagcaacgtgaaagtcatccagatcaacggtagtgcaggtgactatctggatagcctggacatctactatgaacagtacggtaccggcagctcgacccatcgcaagttcggtggtagtggtggaagtccgttctctggtagcggtctgagcagcattgcagttcgtagtggcagctatctggatgcgatcatcattgatggtgtacatcacggtggctctggtggtaacctgagtccgaccttcacctttggatccggtgagtacatcagcaacatgaccattcgtagtggagactacattgacaacatcagctttgaaaccaacatgggtcgtcgctttggtccgtatggtggatctggtggcagtgcaaacaccctgagcaacgtgaaagtcatccagatcaacggtagtgcaggtgactatctggatagcctggacatctactatgaacagtacggcaccggttcttcgacccatcgcaagttcggtggtagtggtggaagtccgttctctggtagcggtctgagcagcattgcagttcgtagtggcagctatctggatgcgatcatcattgatggtgtacatcacggtggctctggtggtaacctgagtccgaccttcacctttggatccggtgagtacatcagcaacatgaccattcgtagtggagactacattgacaacatcagctttgaaaccaacatgggtcgtcgctttggtccgtatggtggatctggtggcagtgcaaacaccctgagcaacgtgaaagtcatccagatcaacggtagtgcaggtgactatctggatagcctggacatctactatgaacagtactaa

Primary structure:

SSTHRKFGGSGGSPFSGSGLSSIAVRSGSYLDAIIIDGVHHGGSGGNLSPTFTFGSGEYISNMTIRSGDYIDNISFETNMGRRFGPYGGSGGSANTLSNVKVIQINGSAGDYLDSLDIYYEQYGTGSSTHRKFGGSGGSPFSGSGLSSIAVRSGSYLDAIIIDGVHHGGSGGNLSPTFTFGSGEYISNMTIRSGDYIDNISFETNMGRRFGPYGGSGGSANTLSNVKVIQINGSAGDYLDSLDIYYEQYGTGSSTHRKFGGSGGSPFSGSGLSSIAVRSGSYLDAIIIDGVHHGGSGGNLSPTFTFGSGEYISNMTIRSGDYIDNISFETNMGRRFGPYGGSGGSANTLSNVKVIQINGSAGDYLDSLDIYYEQY*

4mGRFT

mGRFT-GlyThrGly-mGRFT-GlyThrGly-mGRFT-GlyThrGly-mGRFT

DNA sequence:

agctcgacccatcgcaagttcggtggtagtggtggaagtccgttctctggtagcggtctgagcagcattgcagttcgtagtggcagctatctggatgcgatcatcattgatggtgtacatcacggtggctctggtggtaacctgagtccgaccttcacctttggatccggtgagtacatcagcaacatgaccattcgtagtggagactacattgacaacatcagctttgaaaccaacatgggtcgtcgctttggtccgtatggtggatctggtggcagtgcaaacaccctgagcaacgtgaaagtcatccagatcaacggtagtgcaggtgactatctggatagcctggacatctactatgaacagtacggtaccggcagctcgacccatcgcaagttcggtggtagtggtggaagtccgttctctggtagcggtctgagcagcattgcagttcgtagtggcagctatctggatgcgatcatcattgatggtgtacatcacggtggctctggtggtaacctgagtccgaccttcacctttggatccggtgagtacatcagcaacatgaccattcgtagtggagactacattgacaacatcagctttgaaaccaacatgggtcgtcgctttggtccgtatggtggatctggtggcagtgcaaacaccctgagcaacgtgaaagtcatccagatcaacggtagtgcaggtgactatctggatagcctggacatctactatgaacagtacggtacaggttcttcgacccatcgcaagttcggtggtagtggtggaagtccgttctctggtagcggtctgagcagcattgcagttcgtagtggcagctatctggatgcgatcatcattgatggtgtacatcacggtggctctggtggtaacctgagtccgaccttcacctttggatccggtgagtacatcagcaacatgaccattcgtagtggagactacattgacaacatcagctttgaaaccaacatgggtcgtcgctttggtccgtatggtggatctggtggcagtgcaaacaccctgagcaacgtgaaagtcatccagatcaacggtagtgcaggtgactatctggatagcctggacatctactatgaacagtacggcaccggttcttcgacccatcgcaagttcggtggtagtggtggaagtccgttctctggtagcggtctgagcagcattgcagttcgtagtggcagctatctggatgcgatcatcattgatggtgtacatcacggtggctctggtggtaacctgagtccgaccttcacctttggatccggtgagtacatcagcaacatgaccattcgtagtggagactacattgacaacatcagctttgaaaccaacatgggtcgtcgctttggtccgtatggtggatctggtggcagtgcaaacaccctgagcaacgtgaaagtcatccagatcaacggtagtgcaggtgactatctggatagcctggacatctactatgaacagtactaa

Primary structure:

SSTHRKFGGSGGSPFSGSGLSSIAVRSGSYLDAIIIDGVHHGGSGGNLSPTFTFGSGEYISNMTIRSGDYIDNISFETNMGRRFGPYGGSGGSANTLSNVKVIQINGSAGDYLDSLDIYYEQYGTGSSTHRKFGGSGGSPFSGSGLSSIAVRSGSYLDAIIIDGVHHGGSGGNLSPTFTFGSGEYISNMTIRSGDYIDNISFETNMGRRFGPYGGSGGSANTLSNVKVIQINGSAGDYLDSLDIYYEQYGTGSSTHRKFGGSGGSPFSGSGLSSIAVRSGSYLDAIIIDGVHHGGSGGNLSPTFTFGSGEYISNMTIRSGDYIDNISFETNMGRRFGPYGGSGGSANTLSNVKVIQINGSAGDYLDSLDIYYEQYGTGSSTHRKFGGSGGSPFSGSGLSSIAVRSGSYLDAIIIDGVHHGGSGGNLSPTFTFGSGEYISNMTIRSGDYIDNISFETNMGRRFGPYGGSGGSANTLSNVKVIQINGSAGDYLDSLDIYYEQY*

2mGRFT3

mGRFT-GlyThrGlyGlyThrGlyGlyThrGly-mGRFT

DNA sequence:

agctcgacccatcgcaagttcggtggtagtggtggaagtccgttctctggtagcggtctgagcagcattgcagttcgtagtggcagctatctggatgcgatcatcattgatggtgtacatcacggtggctctggtggtaacctgagtccgaccttcacctttggatccggtgagtacatcagcaacatgaccattcgtagtggagactacattgacaacatcagctttgaaaccaacatgggtcgtcgctttggtccgtatggtggatctggtggcagtgcaaacaccctgagcaacgtgaaagtcatccagatcaacggtagtgcaggtgactatctggatagcctggacatctactatgaacagtacggtaccggcggtacaggtggcaccggttcttcgacccatcgcaagttcggtggtagtggtggaagtccgttctctggtagcggtctgagcagcattgcagttcgtagtggcagctatctggatgcgatcatcattgatggtgtacatcacggtggctctggtggtaacctgagtccgaccttcacctttggatccggtgagtacatcagcaacatgaccattcgtagtggagactacattgacaacatcagctttgaaaccaacatgggtcgtcgctttggtccgtatggtggatctggtggcagtgcaaacaccctgagcaacgtgaaagtcatccagatcaacggtagtgcaggtgactatctggatagcctggacatctactatgaacagtactaa

Primary structure:

SSTHRKFGGSGGSPFSGSGLSSIAVRSGSYLDAIIIDGVHHGGSGGNLSPTFTFGSGEYISNMTIRSGDYIDNISFETNMGRRFGPYGGSGGSANTLSNVKVIQINGSAGDYLDSLDIYYEQYGTGGTGGTGSSTHRKFGGSGGSPFSGSGLSSIAVRSGSYLDAIIIDGVHHGGSGGNLSPTFTFGSGEYISNMTIRSGDYIDNISFETNMGRRFGPYGGSGGSANTLSNVKVIQINGSAGDYLDSLDIYYEQY
